# Biochar from Swine Manure: An Alternative for Nutrient Recovery and Slow-Release Fertilization

**DOI:** 10.1021/acsomega.5c12363

**Published:** 2026-02-25

**Authors:** Larissa Almeida Nascimento, André Pereira Rosa, Augusto Vilela França, Rita de Cássia Superbi de Sousa, Renata Pereira Lopes Moreira

**Affiliations:** † Department of Agricultural Engineering, 28120 Federal University of Viçosa, Viçosa, Minas Gerais 36570-900, Brazil; ‡ Department of Chemistry, 28120 Federal University of Viçosa, Viçosa, Minas Gerais 36570-900, Brazil

## Abstract

The production of swine manure (SM) biochar was optimized to improve the nutrient recovery. SM was activated with KOH, HCl, or MgCl_2_ at different ratios (1:1 and 3:1, v/w) and carbonized at 400, 600, and 800 °C. Results showed that MgCl_2_-activated biochar at 800 °C (3:1) showed enhanced production (84.9%) and the highest phosphate adsorption (2.93 mg g^–1^) and ammonium adsorption (1.27 mg g^–1^). Adsorption followed Langmuir isotherm for phosphate (q_m_ = 67.56 mg g^–1^) and Freundlich for ammonium (q_m_ = 17.48 mg g^–1^). Adsorption kinetics indicated that phosphate uptake was best described by the Elovich model (R^2^ = 0.99), while ammonium followed pseudo-second order kinetics (R^2^ = 0.90), suggesting distinct but predominantly chemisorption-controlled mechanisms. Desorption tests showed a limited solubility of phosphate in water and partial ammonium release, supporting the use of biochar as a slow-release fertilizer. Overall, MgCl_2_-activated biochar at 800 °C proved to be the most effective condition, combining high adsorption capacity with nutrient release potential, highlighting its value for sustainable nutrient recovery in agriculture.

## Graphical abstract

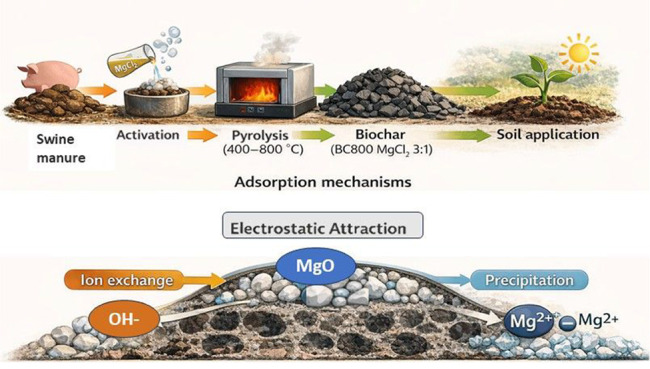

## 1 Introduction

Pork is the most consumed meat worldwide, with a global production of approximately 116.45 million tons in 2024–2025.^1^ This large-scale production generates significant amounts of waste, as swine farming produces between 1.31 and 3.6 kg of manure per animal per day.^2,3^ Swine manure is rich in bioavailable nutrients and is commonly applied to agricultural soils as a fertilizer. However, its continuous use can lead to eutrophication of water bodies, nitrogen volatilization, microbiological contamination, and soil accumulation of potentially toxic metals (e.g., Zn and Cu) and antibiotics.^4^ Therefore, it is important to find alternative ways to recover nutrients.

Thermochemical conversion of animal manure into biochar reduces environmental risks, as the high temperatures involved in the process reduce the bioavailability of metals, eliminate pathogens, and deactivate organic micropollutants and contaminants while minimizing nutrient leaching.^4^ Moreover, biochar exhibits favorable adsorption characteristics, enabling the retention of nutrients from nutrient-rich solutions and wastewaters, with low cost due to the organic matter used as raw material.^5^ After nutrient adsorption, biochar can be reused as a soil amendment or slow-release fertilizer, contributing to sustainable nutrient recycling and reducing environmental impacts.^6^

The physicochemical properties of biochar can vary depending on the raw material, heating rate, temperature, residence time, and the type of reactor used for production.^7^ Furthermore, the activation process can be applied to improve the porous structure of carbon-based materials, such as volume and specific surface area.^8^ Chemical activation is the most common method for modifying biochars, in which chemical agents are used to impregnate the material before or after heat treatment.^9^ Acids, bases, salts (AlCl_3_, MgCl_2_), and metal oxides (MgO, Fe2O_3_) can be used in the process.^10^

In this context, several studies have investigated strategies to enhance nutrient recovery by using modified biochars. However, most of these investigations examine the process within a narrow scope, typically focusing on the adsorption of a single nutrient.^11^ Other studies have assessed the simultaneous removal of nitrogen and phosphorus; however, these investigations generally rely on a single activation route^12^ or a fixed pyrolysis temperature, without emphasizing the nutrient desorption process, which could enable their subsequent use as fertilizers.^13^ In addition, few studies investigate nutrient desorption but focus on only one nutrient^14^ or/and one activating agent.^15^ Understanding these complex interactions is essential for developing efficient nutrient recovery strategies and producing biochar suitable for agricultural reuse as a slow-release fertilizer, which to date has not been reported in the literature.

Therefore, this study aimed to evaluate swine manure as an alternative source of nutrient recovery. Specifically, it sought to optimize the preparation of swine manure-derived biochars for nitrogen and phosphorus removal by assessing different activating agents, activator ratios, and carbonization temperatures. In addition, the adsorption capacity, adsorption kinetics, and nutrient release potential of the optimized biochar were evaluated.

## 2 Material and Methods

### 2.1 Biochar Production

Swine manure (SM) was collected from a pig farm at the Federal University of Viçosa, Minas Gerais, Brazil. The material was oven-dried at 65 °C for 48 h, then ground and sieved to a particle size of 10–60 mesh. Chemical activation was performed by impregnating the SM with the activating agent in the desired proportion (Table 1). The mixtures were dried at 80 °C for 24 h and 105 °C for 3 h, according to the methodology adapted from Li et al.^16^ For biochar production, both pristine and impregnated SM samples were placed in porcelain crucibles, which were covered to limit O_2_ flow while allowing for the partial release of volatiles during pyrolysis.^14^ The crucibles were placed in a muffle furnace (10 °C min^–1^) and held for 1 h at the temperature assigned to each treatment. Subsequently, the biochars were stirred in 0.1 mol L^–1^ HCl solution for 30 min and washed eight times with 500 mL of warm water (50 ± 2 °C) to remove both the activating agent and organic matter residues.^17^ Finally, the samples were dried at 65 °C for 24 h and sieved to 20–115 mesh to ensure homogeneity.

**Table 1:** Experimental Design for Optimizing Biochar Production

Nomenclature	Activating agent	Ratio	Temperature (°C)	Final pH^a^
BC-400	-	-	400	7.54 (±0.04)
BC-600	-	-	600	7.42 (±0.03)
BC-800	-	-	800	7.9 (±0.01)
K-1	KOH	1:1	400	9.00 (±0.03)
K-2	KOH	3:1	400	9.25 (±0.29)
K-3	KOH	1:1	600	10.38 (±0.08)
K-4	KOH	3:1	600	9.1 (±0.25)
K-5	KOH	1:1	800	10.74 (±0.10)
K-6	KOH	3:1	800	8.88 (±0.13)
Cl-1	HCl	1:1	400	7.26 (±0.06)
Cl-2	HCl	3:1	400	6.56 (±0.09)
Cl-3	HCl	1:1	600	7.26 (±0.42)
Cl-4	HCl	3:1	600	6.78 (±0.37)
Cl-5	HCl	1:1	800	7.06 (±0.18)
Cl-6	HCl	3:1	800	7.35 (±0.27)
Mg-1	MgCl_2_	1:1	400	9.58 (±0.18)
Mg-2	MgCl_2_	3:1	400	10.82 (±0.08)
Mg-3	MgCl_2_	1:1	600	11.05 (±0.06)
Mg-4	MgCl_2_	3:1	600	11.29 (±0.11)
Mg-5	MgCl_2_	1:1	800	10.33 (±0.17)
Mg-6	MgCl_2_	3:1	800	10.53 (±0.02)

a pH after 24 h of agitation with ammonium phosphate in the optimization test.

The biochar production conditions were optimized, considering three variables: (i) activating agent: KOH (3 mol L^–1^), HCl (3 mol L^–1^), and MgCl_2_ (3 mol L^–1^); (ii) activating agent ratio: 2:1 and 3:1 (mL of solution: g of manure); (iii) carbonization temperature (400, 600, and 800 °C) (Table 1). The concentrations employed follow the proposals of Nardis et al.^14^ and Li et al.^16^ in order to optimize the magnesium (Mg) content for phosphorus adsorption.

The biochar yield (Y) was calculated after washing the materials, using the following equation:^18^

1 $$Y\left(\%\right)=\frac{M_{b}}{M_{s}}\times 100$$

where M_b_ is the dry mass of biochar produced, and M_s_ is the initial dry mass of biomass.

#### 2.1.1 Optimization of Biochar Production for Nutrient Removal

The response used to evaluate the best conditions for biochar production was the removal of phosphate and ammonia. For this purpose, 0.200 g of each biochar was placed in 125 mL Erlenmeyer flasks, and 50.00 mL of ammonium phosphate solution (50 mg L^–1^) was added. The initial pH was adjusted to 7.2 (±0.2). The mixtures were agitated at 120 rpm for 24 h.^5^ Afterward, suspensions were filtered (0.45 μm) and analyzed for phosphate and ammonia by colorimetric methods (Section 2.6).

The adsorption capacity (Q_e_) of the different biochar materials was calculated by the mass balance, as expressed by eq 2.

2 $$Q_{e}\left(mgg^{-1}\right)=\frac{V\left(C_{i}-C_{e}\right)}{m}$$

#### 2.1.2 Characterization of Biochar

The swine manure was characterized for ash content and elemental and mineral composition. Characterization of the optimized biochar and SM included determination of BET specific surface area (*S*_BET_), pore volume, pore diameter, pH at the point of zero charge (pH_PZC_), Fourier transform infrared spectroscopy (FTIR), and scanning electron microscopy (SEM). The optimized biochar was selected for the isotherm and kinetic experiments.

### 2.2 Batch Adsorption Experiments

#### 2.2.1 Adsorption Kinetics

For adsorption kinetic analyses, optimized biochars and 200 mg L^–1^ solutions of P-phosphate (KH_2_PO_4_) or N-ammonia (NH_4_Cl) were added to a 125 mL Erlenmeyer flask at a solid-to-solution ratio of 4 g L^–1^. The flasks were agitated, and samples were collected at predetermined time intervals (0, 1, 5, 15, 30, 60, 180, 300, 480, 720, and 1,440 min). The samples were immediately filtered (0.45 μm), and the P-phosphate and N-ammonia concentrations were analyzed, as described in Section 2.6. Kinetic models (Table 2) were used to estimate adsorption rates and to support the interpretation of adsorption mechanisms and adsorbent performance. The pseudo-first order (PFO), pseudo-second order (PSO), and Elovich models were fitted to the experimental data.^19^

**Table 2:** Models Were Applied to Describe Adsorption Kinetics

Model	Equation	Model parameters
**PFO**	q_t_ = q_e_ (1 – e^–K_1_t^)	- q_t_ (mg g^–1^): amount of nutrient adsorbed onto the adsorbent per unit mass at time t;
		- q_e_ (mg g^–1^): amount of nutrient adsorbed onto the adsorbent per unit mass at equilibrium;
		- t (min): time;
**PSO**	q_t_ = $\frac{q_{e}^{2}K_{2}t}{1+q_{e}K_{2}t}$	- K_1_ (min^–1^): Pseudo-first order constant;
		- K_2_ (g mg^–1^ min^–1^): Pseudo-second order constant;
**Elovich**	$q_{t}=\frac{1}{b_{E}}\ln\left(1+a_{E}b_{E}t\right)$	- a_E_: initial sorption rate;
		- b_E_ (g mg^–1^): desorption constant.

#### 2.2.2 Adsorption Isotherms

For adsorption isotherm analyses, solutions of P-phosphate (KH_2_PO_4_) at concentrations of 10–350 mg L^–1^ or N-ammonia (NH_4_Cl) at concentrations of 10–300 mg L^–1^ were added to a 125 mL Erlenmeyer flask at a solid-to-solution ratio of 4 g L^–1^. The flasks were agitated for 24 h to ensure an equilibrium. After filtration (0.45 μm), the solutions were analyzed for P-PO_4_ and N-NH_4_, as described Section 2.6. The Langmuir, Freundlich, and Temkin models (Table 3) were fitted to the experimental data.^19^

**Table 3:** Models Applied to the Adsorption Isotherm

Model	Equation	Model parameters
**Langmuir**	$q_{e}=\frac{q_{max}K_{L}C_{e}}{1+K_{L}C_{e}}$	- q_max_ (mg g^–1^): maximum sorption capacity;
		- q_e_ (mg g^–1^): adsorption capacity at equilibrium;
		- C_e_ (mg L^–1^): solution equilibrium concentration;
		- K_L_ (L mg^–1^): Langmuir constant;
**Freundlich**	q_e_ = K_F_C_e_^1/n^	- K_F_ ((mg g^–1^) (mg L^–1^) ^(−1/n)^): Freundlich constant;
		- n: constant related to the adsorption strength of adsorbent;
**Temkin**	$q_{e}=\frac{RT}{b_{T}}\ln\left(A_{T}C_{e}\right)$	- R (8.314 J mol^–1^ K^–1^): universal gas constant;
		- T (K): absolute temperature;
		- A_T_ (L mg^–1^): Temkin equilibrium constant;
		- b_T_ (J mol^–1^): adsorption energy.

### 2.3 Volatilization

NH_3_ volatilization from biochars was evaluated. Samples of 0.100 g of biochar and 25.00 mL of NH_4_Cl solution (5, 100, and 200 mg L^–1^) were added to 120 mL penicillin vials and then sealed with rubber stoppers and aluminum seal. After 24 h of agitation (190 rpm), 40 mL of headspace gas was collected and immersed in H_3_BO_3_ to capture NH_3_. The trapped NH_3_ was measured as N-NH_4_^+^ using a colorimetric method, and the N-NH_4_^+^ standards were prepared by using 1% H_3_BO_3_ matrix.^20^ Control assays were performed under the same conditions but without biochar addition. This experiment was done in duplicate and at room temperature (∼25 °C).

### 2.4 Desorption Test

A desorption study was carried out to evaluate the potential of biochar as a slow-release fertilizer. A batch experiment was conducted in 125 mL Erlenmeyer flasks containing 50 mL of extractant solutions and 0.200 g of biochar previously saturated with ammonium phosphate. A 2 mol L^–1^ KCl solution was used as an extractant for NH_4_^+^ recovery,^15^ Mehlich-I (0.025 mol L^–1^ H_2_SO_4_ + 0.05 mol L^–1^ HCl) and 2% citric acid solution for ortho-P, and deionized water was employed for both nutrients.^14^ The flasks were agitated at room temperature for 24 h, and after filtration (0.45 μm), the remaining concentrations of P-PO_4_ and N-NH_4_ were analyzed. Controls, without the saturation step, were conducted to evaluate the inherent release of phosphorus and nitrogen from the material.

### 2.5 Statistical Analysis

Production yield (Y) and adsorption capacity data obtained in the optimization test were subjected to normality analysis using the Shapiro-Wilk test and homogeneity of variance analysis using the Bartlett test, followed by analysis of variance (ANOVA). The means were compared using the Tukey test with a 5% significance level. The statistical analysis was performed using R software, version 4.5.0.

### 2.6 Analytical Methods

Ash content was determined using the standard method ASTMD 1762-84.^21^ FTIR spectra was performed on a VARIAN 660 FTIR instrument equipped with a GladiATR. A carbon sample was added to the diamond crystal of the GladiATR, and spectral readings were taken for the 4000 to 400 cm^–1^ range. For X–ray diffraction (XRD) analysis, measurements were conducted using a diffractometer (Bruker D8–Discover, Germany) equipped with a copper tube and Goebel mirror, using Ni–filtered Cu–Kα radiation (λ = 1.5418 Å). Scanning was performed at a rate of 0.05° s^–1^ over a 2θ range of 5° to 90°. The MATCH software (v 3.8.3.151, trial version) was used to identify XRD peaks, bands, and spectral signatures.

To determine the point of zero charge (pH_PZC_), 30 mL of a 0.01 M NaCl solution was prepared in several flasks, and the initial pH of each was adjusted between 2 and 12. Subsequently, 0.15 g of the biochar produced at 800 °C and activated with MgCl_2_ (3:1) was added to each flask. After 24 h of agitation, the final pH values were recorded. The pH_PZC_ was identified as the intersection point between the pH_final_ versus pH_initial_ curve and the bisector line.^22^

The optimized biochar and swine manure were chemically analyzed. Total phosphorus was determined by a colorimetric method after nitroperchloric digestion. Total nitrogen was measured by the Semi-Micro-Kjeldahl method.^23^ Specific surface area was determined using Nova 600 Series equipment (Anton Paar). The analysis was performed based on N_2_ adsorption–desorption isotherms, using the Brunauer, Emmett, and Teller (BET) method to determine the surface area, and the Barrett–Joyner–Halenda (BJH) method to evaluate the pore size distribution.

Scanning electron microscopy (SEM) images were obtained using JEOL equipment (JSM-6010LA), with a resolution of 4 nm (with a 20 kV beam). The samples were pretreated with gold metallization (Quorum Q150R S Metallizer).

Nutrient concentrations (ammonium and phosphate ions) were measured colorimetrically. The N-NH_4_^+^ content was determined by Hood-Nowotny et al.,^24^ and the ortho-P content was determined by the ascorbic acid method.^23^

## 3 Results and Discussion

### 3.1 Influence of Preparation Conditions on Biochar Yield

Biochar yield results are listed in Figure 1. Biochar yield decreased as the pyrolysis temperature increased for all activating agents. Lower pyrolysis temperatures resulted in higher biochar yields because thermal degradation and volatilization of organic matter were less extensive, leading to reduced mass loss.^25^ The use of MgCl_2_ enhanced the biochar yield, which can be attributed to its ability to inhibit depolymerization reactions. Furthermore, increasing the MgCl_2_ concentration resulted in higher yields, likely due to the reduction in noncondensable gas production.^26^ In contrast, biochars activated with KOH and HCl exhibited lower yields, being statistically equal to or lower than those of the corresponding unmodified biochars. These results can be attributed to the higher efficiency of these agents in removing volatile matter,^10^ promoting a reduction in biochar yield.

**Figure 1:**
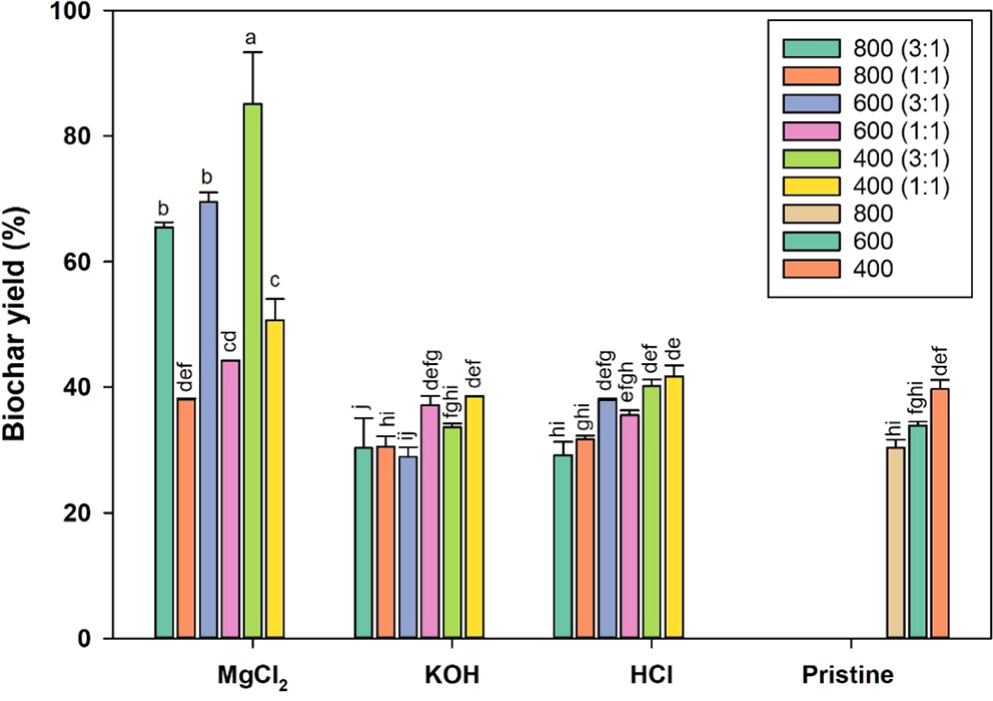
Biochar yield (%) of swine manure biochars prepared under different conditions. Legend: Means followed by the same letter do not differ at the 5% level (Tukey test).

### 3.2 Effect of Preparation Conditions on Nutrients Removal

The adsorption results for phosphate and ammonium by the different biochars are presented in Tables 4 and 5.

**Table 4:** Adsorption Capacity (mg g^–1^) of Phosphorus (P-PO_4_^3–^) of Modified Swine Manure Biochars (HCl, KOH, MgCl_2_) Produced at 400, 600, and 800 °C^a^,^b^

		Temperature (°C)		
Activating agent	Ratio	400	600	800
HCl	1:1	–2.34^c^ (±0.00) Cb*b*	–1.61^c^ (±0.10) Bb*b*	–1.16^c^ (±0.05) Ab*c*
	3:1	–1.98^c^ (±0.04) Ca*c*	–1.15^c^ (±0.09) Ba*c*	–0.60^c^ (±0.02) Aa*c*
KOH	1:1	0.48 (±0.01) Ab*a*	–3.30^c^ (±018) Cb*c*	–0.06^c^ (±0.01) Bb*b*
	3:1	2.71 (±0.02) Aa*a*	1.45 (±0.02) Ba*b*	2.73 (±0.03) Aa*b*
MgCl_2_	1:1	0.64 (±0.06) Bb*a*	2.55 (±0.13) Ab*a*	2.59 (±0.02) Ab*a*
	3:1	2.25 (±0.00) Ba*b*	2.83 (±0.01) Aa*a*	2.93 (±0.05) Aa*a*

a Average (± standard deviation).

b Values followed by the same uppercase letter within temperature levels, lowercase letters within ratio levels, and lowercase letters in italics within activating agent levels do not differ significantly according to Tukey’s test at a 5% significance level.

c The negative values result from the release of mineral phosphorus present in the biochars, as shown in Table S1.

**Table 5:** Adsorption Capacity (mg g^–1^) of Nitrogen (N-NH_4_^+^) of Modified Swine Manure Biochars (HCl, KOH, MgCl_2_) Produced at 400, 600, and 800 °C^a^,^b^

		Temperature (°C)		
Activating agent	Ratio	400	600	800
HCl	1:1	1.07 (±0.04) Aa*a*	0.27 (±0.02) Ba*b*	0.35 (±0.03) Ba*c*
	3:1	0.10 (±0.01) Bb*c*	0.31 (±0.03) ABa*b*	0.52 (±0.03) Aa*b*
KOH	1:1	0.57 (±0.12) Ba*b*	0.97 (±0.05) Aa*a*	0.77 (±0.00) ABa*b*
	3:1	0.39 (±0.03) Ba*b*	0.68 (±0.08) Ab*a*	0.41 (±0.01) Bb*b*
MgCl_2_	1:1	0.57 (±0.06) Cb*b*	0.89 (±0.02) Ba*a*	1.51 (±0.29) Aa*a*
	3:1	0.79 (±0.13) Ba*a*	0.69 (±0.04) Bb*a*	1.27 (±0.08) Ab*a*

a Average (±standard deviation).

b Values followed by the same uppercase letter within temperature levels, lowercase letters within ratio levels, and lowercase letters in italics within activating agent levels do not differ significantly according to Tukey’s test at a 5% significance level.

The preparation conditions significantly influenced the adsorption of both nutrients. For phosphate, MgCl_2_-activated biochar at a 3:1 ratio prepared at 800 °C exhibited the highest adsorption. In contrast, unactivated biochars and those activated with HCl exhibited greater phosphorus release compared to their adsorption capacity (C_e_ > C_O_, eq 2), resulting in negative adsorption values, as confirmed by the release test in deionized water (see Supporting Information, Table S1). Negative phosphorus (P-PO_4_^3–^) removal rates were also found by Ji et al.^5^ and Luo et al.^12^ KOH-activated biochars exhibited variable phosphorus adsorption, with positive values comparable to MgCl_2_-treated biochars in some treatments and negative values in others.

Regarding ammonium, the highest adsorption was observed for MgCl_2_-activated biochars at a 1:1 ratio prepared at 800 °C (54% efficiency). HCl-activated and unactivated biochars showed the lowest adsorption, while KOH-activated biochars exhibited intermediate performance. Considering both phosphate and ammonium, the MgCl_2_-activated biochar at a 3:1 ratio and produced at 800 °C was, therefore, selected for subsequent experiments.

### 3.3 Characterization

The raw swine manure was characterized prior to biochar production. Proximate analysis was performed to determine moisture, volatile matter, and ash contents, which were 5.67%, 69.20%, and 26.50%, respectively (Table S2). The relatively high ash content reflects the presence of inorganic constituents typical of swine manure. Elemental and mineral composition of the raw material was assessed by energy-dispersive X-ray spectroscopy (EDS), revealing the presence of C, O, N, P, K, and Ca (see Figure S3). This mineral-rich matrix is a characteristic feature of swine manure, as supported by literature.^12^

Fourier transform infrared spectroscopy (FTIR) was used to identify the main functional groups present in swine manure, modified and unmodified biochars (Figure 2).

**Figure 2:**
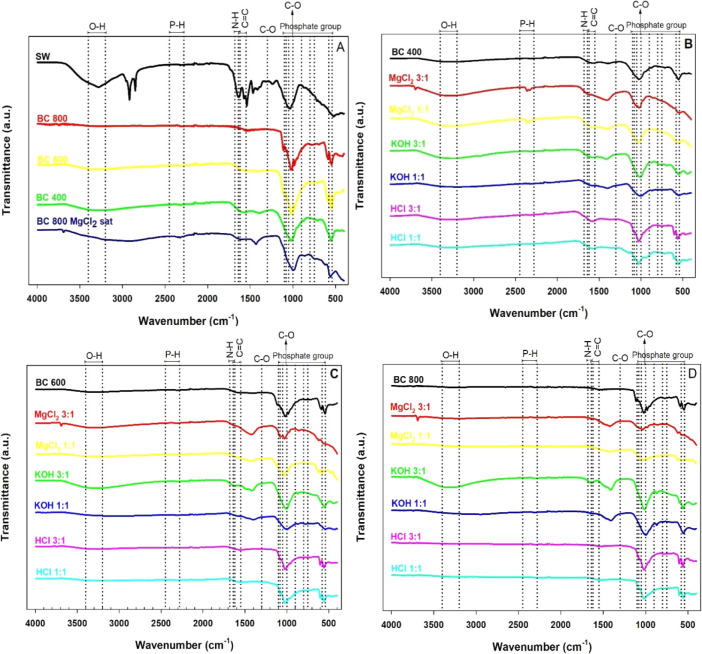
FTIR spectra of raw swine manure (SM) and derived biochars produced at 400, 600, and 800 °C, before and after chemical activation (MgCl_2_, KOH, HCl; 1:1 and 3:1). Legend: (A) SM and unmodified biochars (400–800 °C), including MgCl_2_-saturated biochar; (B–D) activated biochars at 400, 600, and 800 °C.

FTIR spectra (Figures 2A–D) show the structural evolution of swine manure (SM) into biochars as well as the effects of chemical activation. In SM (Figure 2A), a broad band is observed at ∼3400 cm^–1^, attributed to hydroxyls (O–H), in addition to signals at 2920 and 2854 cm^–1^ (C–H of aliphatic bonds), 1640–1550 cm^–1^ (N–H and C=O of amines and carboxylic acids), and phosphate groups.^27^ In pyrolyzed biochars, especially at 800 °C, these bands decrease in intensity or disappear, indicating more intense carbonization and a reduction of surface oxygenated groups.

Among the activated materials (Figures 2B–D), treatments with MgCl_2_, especially in a 3:1 ratio, showed greater intensity in the 1000–500 cm^–1^ region, associated with C–O and P–O vibrations, suggesting phosphorus stabilization in the biochar matrix.^28,29^ Activation with KOH intensified bands at 1100–1000 cm^–1^, attributed to oxygenated structures (ethers, phenols), in addition to the presence of carboxylate group stretching (∼1420 cm^–1^), indicating the formation of carboxylic acid salts at intermediate temperatures (400–600 °C).^27^ On the other hand, biochars activated with HCl showed spectra with lower intensity of functional groups, consistent with the removal of compounds during acid activation.

The spectrum of MgCl_2_ biochar (3:1, 800 °C) saturated with ammonium phosphate (Figure 2A) showed an increase in the band at ∼1040 cm^–1^, characteristic of phosphate,^28,29^ corroborating the adsorption data.

In the case of activation with HCl, the reduction of functional groups and the final pH of the optimization (Table 1) close to neutrality restrict the formation of precipitates, which may justify the lower adsorption efficiency. In biochars activated with KOH, there may be a reduction in the concentration of the main exchangeable cations (Mg^2+^, Ca^2+^, Na^+^, and K^+^) due to their solubilization in the KOH solution.^10^ This decrease limits the availability of active sites for ion exchange reactions, which may contribute to the lower efficiency and variability observed in the adsorption of phosphorus and ammonia in some KOH treatments.

In MgCl_2_-activated biochars, the MgCl_2_ deposited on the biomass is decomposed into MgO at high temperatures during pyrolysis.^10^ In aqueous solutions, H^+^ is consumed by MgO, causing the increase in the pH of the medium observed at the end of the optimization. Under high pH conditions, the precipitation of calcium phosphates and the generation of soluble mineral phases based on potassium, magnesium, or nitrogen can contribute significantly to phosphorus sorption mechanisms.^14^

The XRD patterns of the swine manure and MgCl_2_-modified biochar produced at 800 °C are presented in Figure 3.

**Figure 3:**
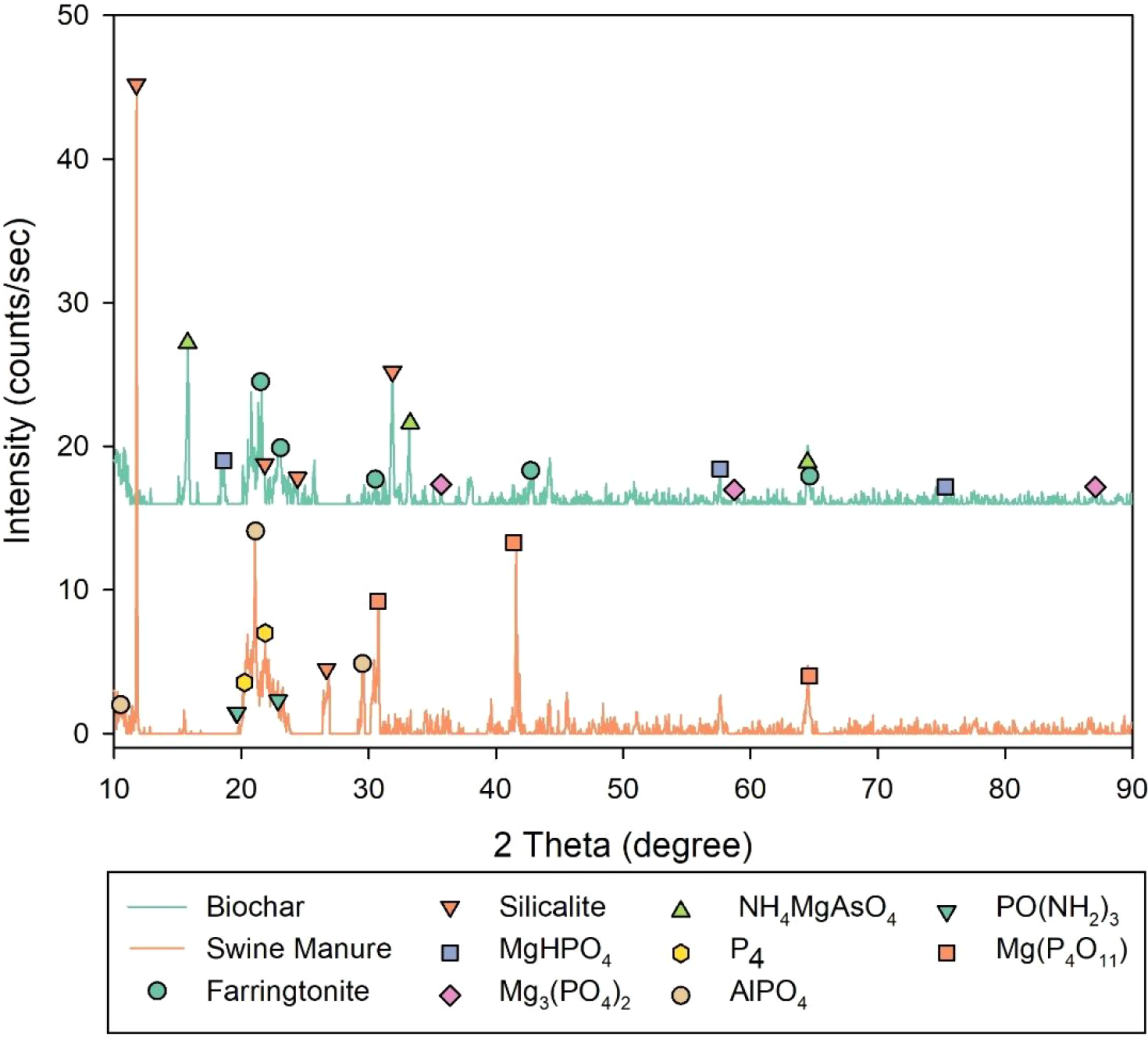
XRD patterns of swine manure and saturated Mg-modified biochar.

SM revealed the presence of crystalline peaks associated mainly with silicalite (SiO_2_), aluminum phosphate, magnesium ultraphosphate (MgP_4_O_11_), and phosphoric triamide (PO­(NH_2_)_3_. In turn, the saturated biochar exhibited phosphate crystals, such as farringtonite, magnesium phosphate (Mg_3_(PO_4_)_2_), newberyite (MgHPO_4_·3H_2_O), and arsenstruvite (NH_4_MgAsO_4_·6H_2_O). Moreover, the predominance of silicalite indicates that a portion of the mineral matrix remained thermally stable after high-temperature carbonization. These findings support a dual mechanism for nutrient retention: (i) adsorption of phosphate and ammonium onto oxygenated functional groups and Mg-enriched surface sites, followed by (ii) localized precipitation of crystalline Mg–P–N phases.

The 800 MgCl_2_ 3:1 biochar obtained a pH_PZC_ equal to 11.2 (Figure 4). The point of zero charge (PZC) of an adsorbent is that at which the pH value corresponds to a net surface charge equal to zero.^30^ Thus, in solutions with a pH higher than the pH_PZC_, the surface charge is negative, and the adsorption of cations is favored, while at pHs lower than the pH_PZC_, the surface charge is positive, and the adsorption of anions is favored. Phosphorus has the ionic form H_2_PO_4_^2–^ predominant between pH 7 and 12.^31^ In this context, the adsorption of phosphorus would be favored by the electrostatic attraction within the pH range found in the optimization (10.53) (Table 1).

**Figure 4:**
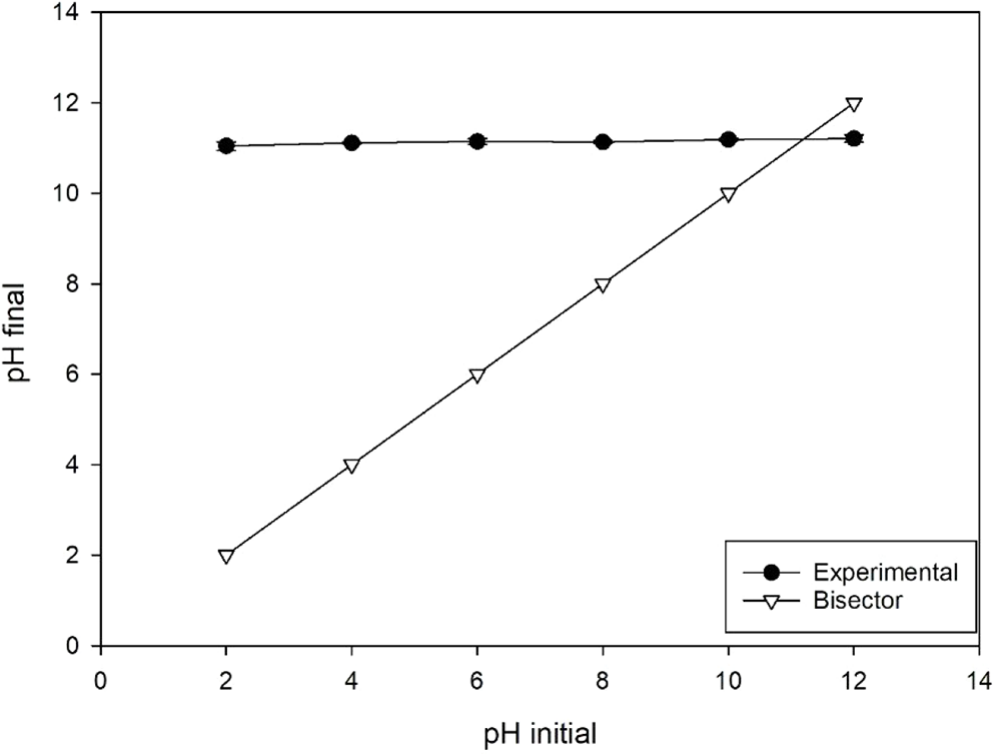
pH at the point of zero charge of Mg-modified biochar.

The chemical characterization data for biochar and swine manure are presented in Table 6. The phosphorus concentration in biochar was higher than in swine manure, resulting from the enrichment of P during pyrolysis due to its non-volatile nature.^4^ Furthermore, the phosphorus content measured in saturated biochar reflected both the inherent phosphorus originally present in the material and the phosphorus subsequently adsorbed during the saturation step. In contrast, the total nitrogen content of the biochar decreased compared to the precursor material. This loss of nitrogen is attributable to thermal decomposition pathways that produce volatile N-species (e.g., NH_3_, HCN, and HNCO), whose relative yields depend strongly on pyrolysis temperature, heating rate, and feedstock N-chemistry.^32^

**Table 6:** Chemical Characterization of Swine Manure (SM), Biochar (BC 800 MgCl_2_ 3:1), and Saturated Biochar (S BC 800 MgCl_2_ 3:1)^a^

Parameter	SM	BC 800 MgCl_2_ 3:1	S BC 800 MgCl_2_ 3:1
Total P (mg g^–1^)	28.96 ± 1.30	33.34 ± 4.79	86.96 ± 11.72
Total N (mg kg^–1^)	49.77 ± 2.14	10.23 ± 0.80	6.25 ± 0.31

a Mean ± standard deviation.

The results of the pore structure analysis are presented in Table 7 and Figure 5. The thermal treatment at 800 °C with MgCl_2_ activation promoted a 25% increase in surface area compared to that of the SM. Furthermore, the pore volume was seven times greater in the modified biochar compared to that of the SM, indicating a lighter and more porous material. In addition, both isotherms were classified as type IVa, indicating mesoporous materials. In fact, the average pore diameter was 3.8 nm for both samples, a value that falls within the mesopore range.^33^ However, the pore distribution (BJH-Desorption-dV­(logd)) and the presence of hysteresis in the isotherm suggest a greater presence of macropores in the SM, while in the biochar there is a structure of meso- and micropores.^33^ In summary, the data demonstrate that activation with MgCl_2_, combined with pyrolysis at 800 °C, was effective in generating a material with a high surface area and a highly porous structure.

**Table 7:** Porosity and Surface Area Properties of SM and BC 800/MgCl_2_ 3:1

Parameter	Swine manure	BC 800 °C MgCl_2_
BET surface area (m^2^ g^–1^)	1.83	46.85
Microporous area (m^2^ g^–1^)	0.20	24.41
Total pore volume (cm^3^ g^–1^)	0.0079	0.0573
Average pore diameter (nm)	3.85	3.88

**Figure 5:**
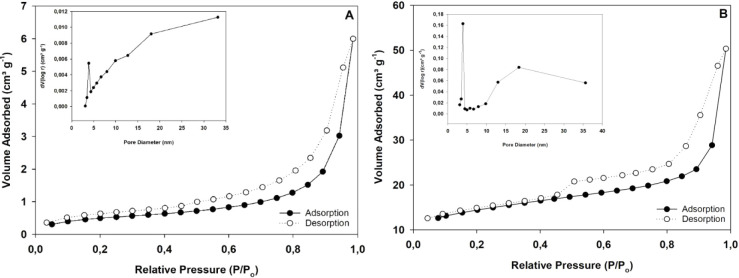
N_2_ adsorption/desorption isotherms for (A) SM—swine manure, (B) BC 800 MgCl_2_ 3:1—biochar modified with MgCl_2_ (3M) in a 3:1 ratio, produced at 800 °C.

The surface morphology of optimized biochar and swine manure was observed by using SEM analysis, with images presented in Figure 6. The morphological analysis of swine manure revealed a predominantly compact and relatively smooth surface. In contrast, the biochar activated with MgCl_2_ and produced at 800 °C showed a fragmented surface with smaller particles.

**Figure 6:**
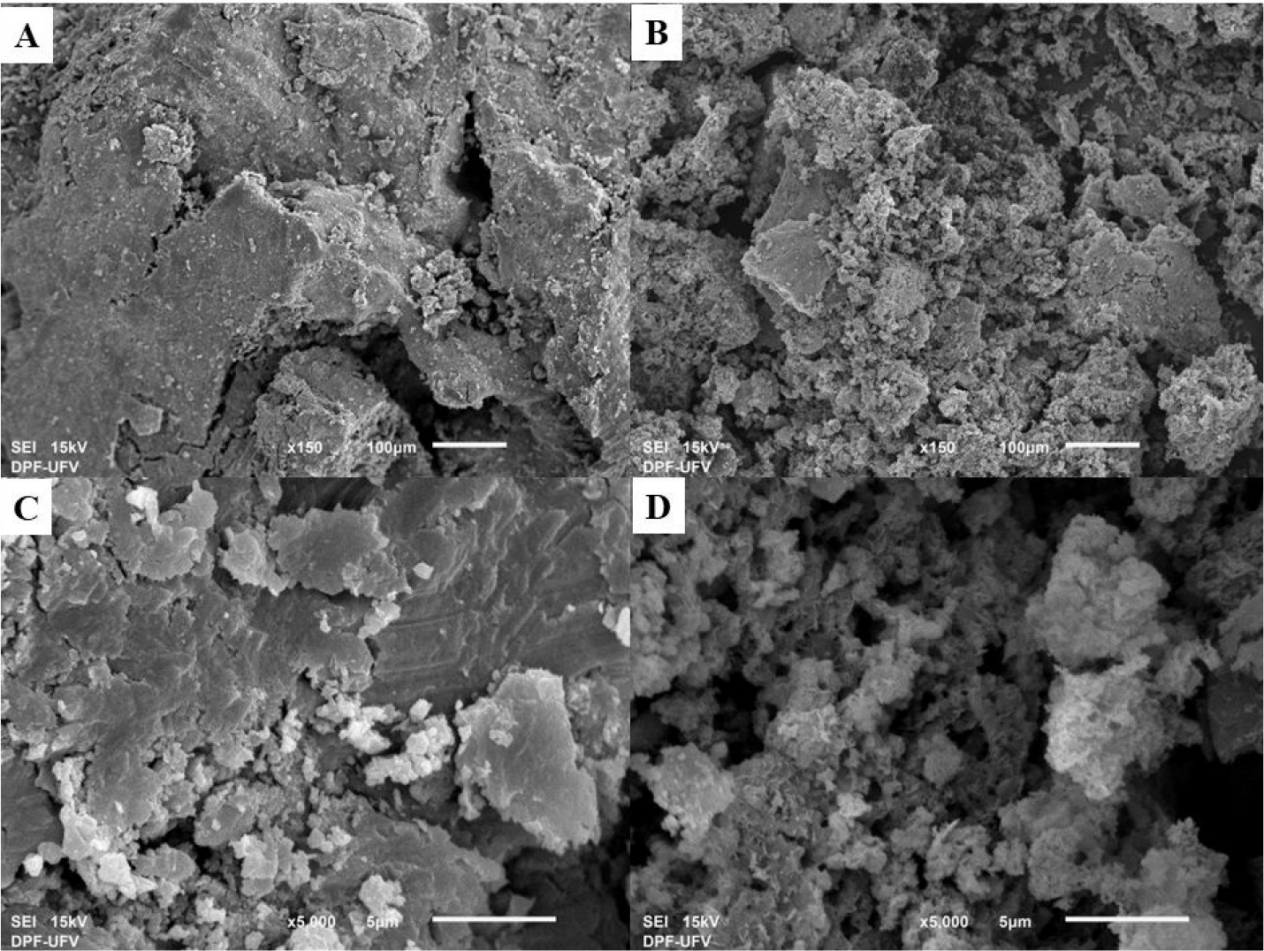
SEM images of SM (A, C) and BC800/MgCl_2_ (B, D).

### 3.4 Adsorption Kinetics

The adsorption kinetics of phosphate (P-PO_4_^–3^) and ammonium (N-NH_4_^+^) ions on BC 800 MgCl_2_ (3:1) biochar were evaluated using pseudo-first order (PFO), pseudo-second order (PSO), and Elovich models (Figure 7, Table 8). For phosphorus, the Elovich model provided the best fit to the experimental data (R^2^ = 0.99), suggesting that the adsorption of phosphate occurs on a highly heterogeneous surface.^19^ The initial adsorption rate (a_E_) for P was 26.01 mg g^–1^ h^–1^, while the low desorption constant (b_E_ = 0.18 g mg^–1^) indicates that the phosphate ions are strongly retained.

**Figure 7:**
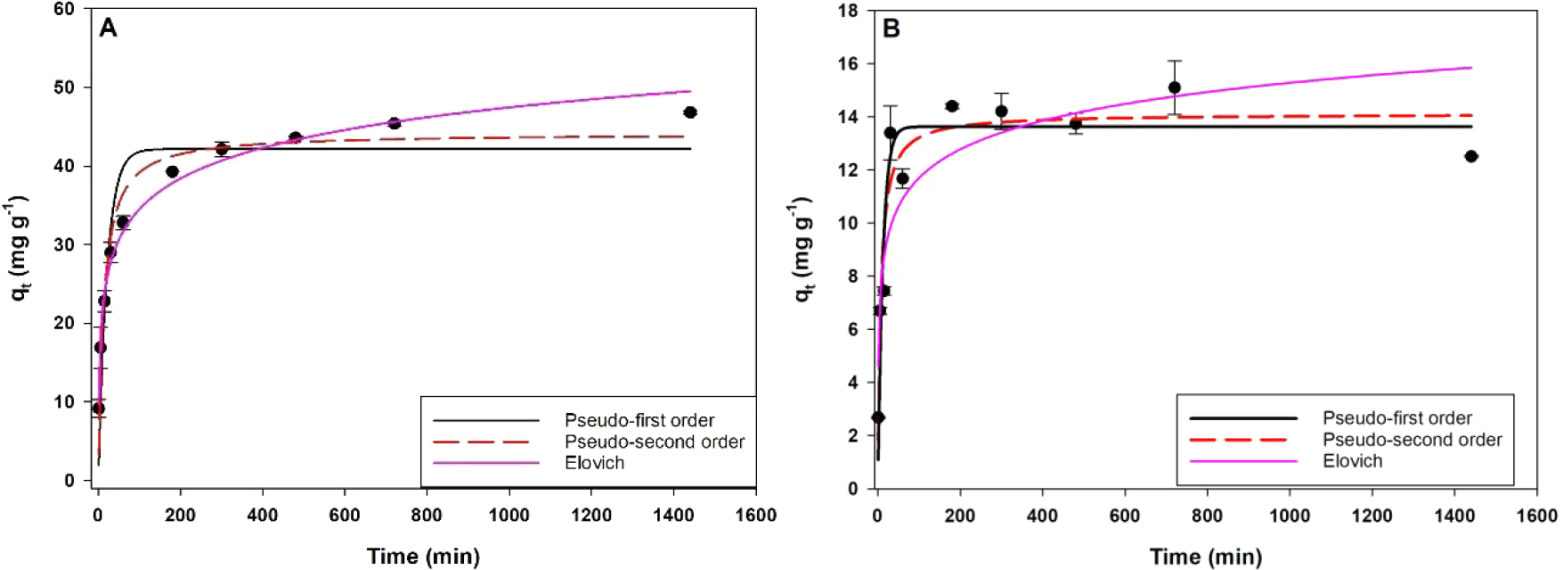
Phosphate (A) and ammonia (B) adsorption kinetics of BC 800 MgCl_2_ 3:1 biochar.

**Table 8:** Phosphate and Ammonia Kinetic Parameters for BC 800 MgCl_2_ 3:1 Biochar

		Adsorbate	
Model	Parameters	P-PO_4_	N-NH_4_
PPO	q_e_ (mg g^–1^)	42.15	13.63
	K_1_ (h^–1^)	0.048	0.083
	R^2^	0.858	0.867
PSO	q_e_ (mg g^–1^)	44.17	14.12
	K_2_ (g(mg h)^−1^)^−1^	0.002	0.011
	R^2^	0.94	0.90
Elovich	a_E_	26.01	28.78
	b_E_	0.18	0.64
	R^2^	0.99	0.78

For ammonium (N–NH_4_^+^), the Elovich model showed a higher b_E_ (0,64 g mg^−1^), indicating a less stable adsorption, and the lower R^2^ for ammonium (0.78), suggesting a more uniform interaction compared to phosphate. In turn, the PSO model showed the highest correlation (R^2^ = 0.90), indicating that the rate-limiting step is predominantly governed by chemisorption.^34^ Although the adsorption rate constant (K_2_) was higher for ammonium (0.0105 g mg^–1^ h^–1^), indicating faster adsorption, phosphate showed a higher retention capacity. These results highlight the potential of MgCl_2_-modified biochar as an effective adsorbent for the simultaneous removal of nutrients in aqueous solutions.

### 3.5 Adsorption Isotherms

The adsorption isotherms of P–PO_4_^–3^ and N–NH_4_^+^ onto BC 800 MgCl_2_ 3:1 biochar were evaluated using the Langmuir, Freundlich and Temkin models (Figure 8, Table 9). For phosphorus, the Langmuir model showed the best fit to the experimental data (R^2^ = 0.97), indicating that adsorption occurs predominantly as monolayer coverage on a homogeneous surface.^35^ The maximum adsorption capacity (q_m_) estimated by the model was 67.56 mg g^–1^, and the Langmuir constant (K_L_) was 0.37 L mg^–1^, reflecting the affinity of the biochar for phosphate ions.^36^ The q_m_ value is within the range reported in the literature for Mg-modified feedstocks: 27.3–138 mg g^–1^ for lignocellulosic materials,^37,38^ 50 mg g^–1^ for sewage sludge,^39^ and 0.86–324.17 mg g^–1^ for swine manure-derived biochars.^5,14^ Differences in q_m_ between studies are often attributable to feedstock composition, the nature and amount of the activating/loading activating agent, impregnation protocol, and pyrolysis parameters (temperature, heating rate, and residence time).

**Figure 8:**
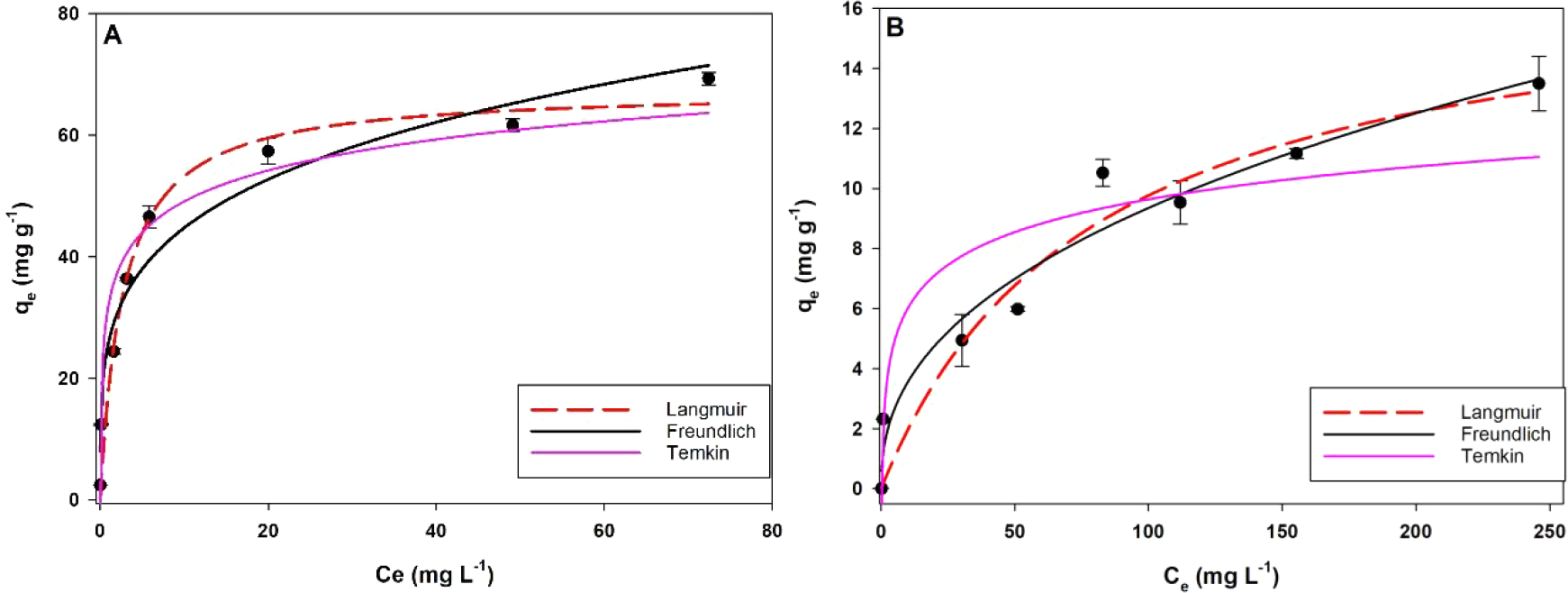
Phosphate (A) and ammonia (B) adsorption isotherms of biochar BC800 MgCl_2_ 3:1.

**Table 9:** Phosphate and Ammonia Isotherm Parameters for BC 800 MgCl_2_ 3:1 Biochar

		Adsorbate	
Model	Parameters	P-PO_4_	N-NH_4_
Langmuir	q_m_ (mg g^–1^)	67.56	17.48
	K_L_ (L mg^–1^)	0.37	0.0126
	R^2^	0.97	0.9436
Freundlich	K_F_ ((mg g^–1^) (mg L^–1^) ^(−1/n)^)	26.01	1.35
	1/n	0.2362	0.42
	R^2^	0.96	0.95
Temkin	A_T_ (L mg^–1^)	79.99	4.5608
	b_T_ (J mol^–1^)	337.305	1574.41
	R^2^	0.9397	0.8468

In contrast, the maximum adsorption capacity for N–NH_4_^+^ was substantially lower than that observed for phosphorus (q_m_ = 17.48 mg g^–1^), and the Langmuir constant (K_L_) was 0.0126 L mg^–1^, indicating weaker affinity for the active sites. q_m_ values were between 1.54 ^5^ and 50 mg g^–1^.^37^ In this case, the Freundlich model provided a better fit (R^2^ = 0.9549) compared with the Langmuir model (R^2^ = 0.9436). The Freundlich constant (K_F_) was 1.35, confirming the lower adsorption capacity for ammonium relative to that for phosphate. The 1/n value, related to surface heterogeneity and adsorption intensity, was higher for ammonium (1/n = 0.42), suggesting lower reversibility and greater heterogeneity of the adsorption sites.^36,40^

Temkin model was also applied to investigate the interaction potential between the adsorbates and the biochar. For phosphorus, the Temkin constant (b_T_) was 337.30 J mol^–1^. This relatively low value for the heat of adsorption is characteristic of physical adsorption or weak electrostatic interactions between the phosphate anions and the magnesium-functionalized surface. Furthermore, the high equilibrium binding constant (A_T_ = 79.99 L mg^–1^) indicates a strong initial affinity^40^ of the biochar for P–PO_4_^–3^ ions, corroborating the high K_L_ value obtained in the Langmuir model.

For ammonium, the Temkin model presented the lowest correlation among the three models (R^2^ = 0.8468). The calculated b_T_ value was 1574.41 J mol^–1^, which is higher than that observed for phosphorus, suggesting that although the total capacity for nitrogen is lower, the change in adsorption heat during the process is more pronounced for ammonium ions. Additionally, the A_T_ value for ammonium (4.56 L mg^–1^) was approximately 17 times lower than that of phosphate, further confirming the weaker affinity of the active sites for N–NH_4_^+^.

### 3.6 Volatilization

The results of the ammonia volatilization are shown in Figure 9. Volatilization measurements indicated generally low NH_3_ losses across treatments (<1.8% of initial N), but statistical analysis showed a significant difference between biochar and control only at the lowest ammonium concentration (5 mg L^–1^; *p* < 0.05). At 100 and 200 mg L^–1^, volatilization rates did not differ significantly between biochar-treated and control samples (*p* > 0.05).

**Figure 9:**
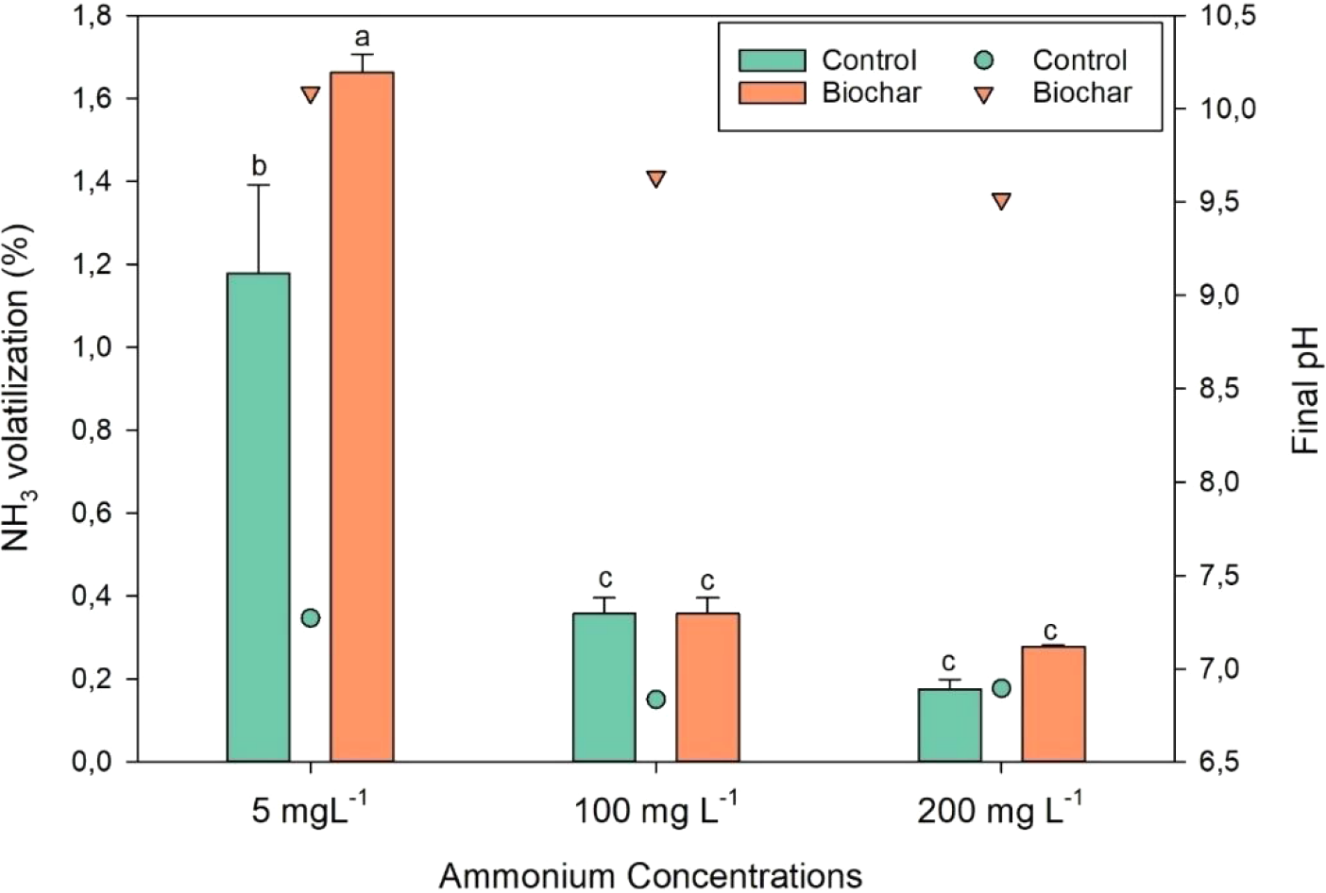
Ammonia volatilization (%) and final pH at different ammonium concentrations in treatments with and without (control) biochar addition. Legend: Bars represent mean values (±SD). Different letters above the bars indicate significant differences between treatments at the same ammonium concentration according to Tukey’s test (*p* < 0.05). Points indicate the final pH of each treatment.

The concentration-dependent pattern can be explained by two complementary mechanisms. First, the solutions containing biochar exhibited higher final pH values (9.51–10.08), which shifts the NH_4_^+^ ⇌ NH_3_ equilibrium toward free NH_3_ and can enhance volatilization under dilute conditions. Second, at higher ammonium concentrations (100–200 mg L^–1^) the biochar favors the formation of less volatile species (e.g., surface-associated Mg–phosphate/struvite-like phases), reducing the fraction available for conversion to NH_3_.^41^ Taken together, these processes explain why volatilization differences are significant only at the lowest concentration.

Banik et al.^15^ reported NH_3_ losses between 0.006 and 1.9% in experiments with biochars, while control treatments showed higher emissions (up to 4.5%). In their study, the lower volatilization in the presence of biochar was associated with microbial activity and localized pH. Importantly, the absolute volatilization losses were small in all cases, indicating that the majority of nitrogen removal observed in the adsorption assays is attributable to uptake by the biochar rather than gaseous losses.

### 3.7 Desorption

The desorption analysis of phosphorus and nitrogen from MgCl_2_-modified biochar highlights the potential of these materials as slow-release fertilizers. Phosphorus desorption (extraction) was performed using deionized water, 2% citric acid, and a Mehlich-1 solution (Figure 10).

**Figure 10:**
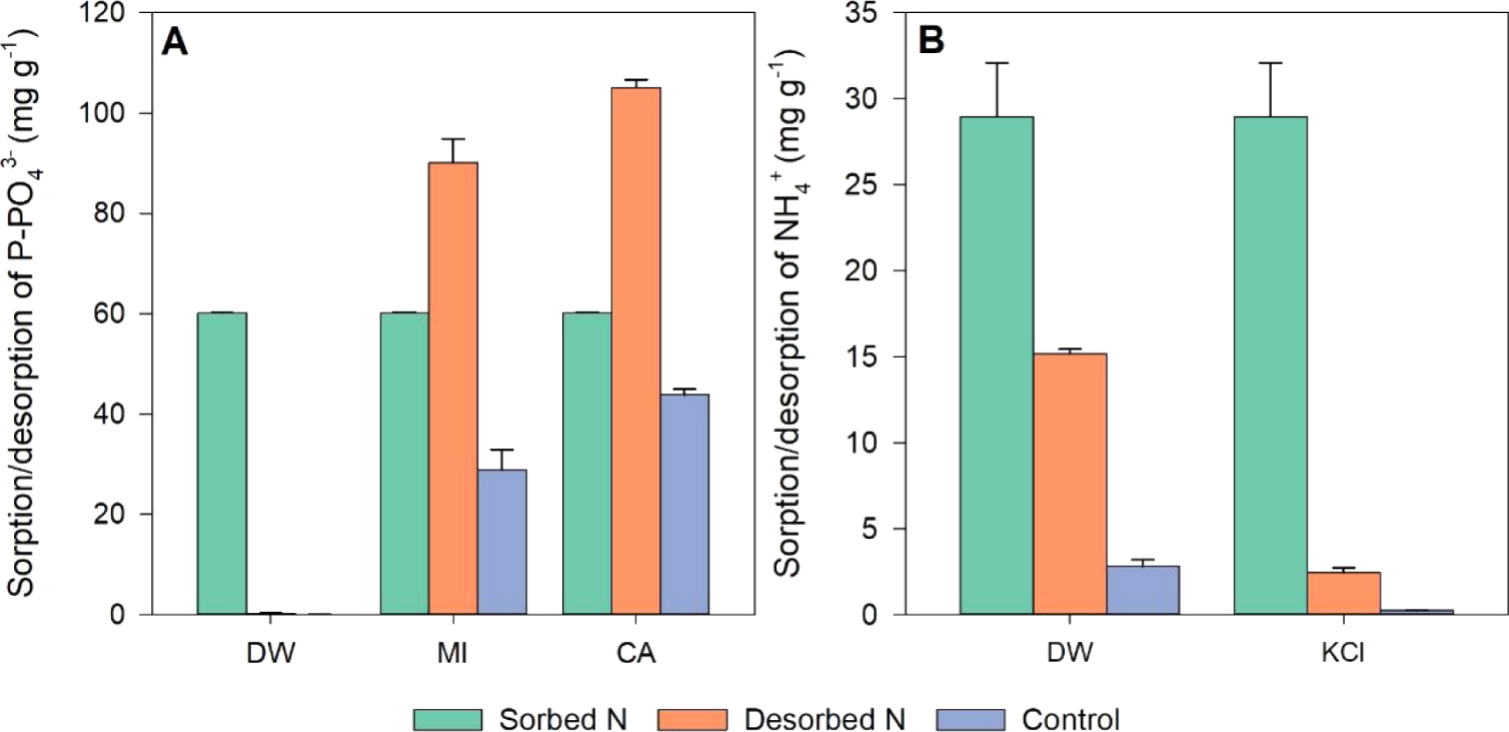
Desorption (%) of phosphorus (A) and ammonium ion (B) using different extractants. Legend: DW: deionized water, M1: Mehlich-1, CA: 2% citric acid, and KCl: 2 M KCl. Control treatments were performed without the saturation step.

Water-soluble P represents the fraction readily available for plant uptake and is easily leached in sandy soils or fixed in clayey soils rich in Fe and Al oxides. The P extracted with Mehlich-1 and citric acid reflects the chemically soluble species potentially available to plants through rhizosphere exudates.^42^ Negligible recovery in water indicates that phosphorus is retained, possibly through precipitation with magnesium or stable electrostatic interactions. In contrast, Mehlich-1 and citric acid desorption exceeded 100%, indicating not only the release of previously adsorbed phosphorus but also the dissolution of structural P from the biochar matrix under acidic conditions. This result can be attributed to the nature of the chemical interactions involved in adsorption, indicating that in these materials phosphorus was predominantly adsorbed through electrostatic interactions or reversible ion exchange.

Ammonium ion (NH_4_^+^) desorption exhibited greater release in deionized water than in a 2 M KCl solution. This result suggests that nitrogen is retained mainly through chemical mechanisms such as precipitation in the form of struvite (NH_4_MgPO_4_·6H_2_O) or interaction with magnesium phosphates. During KCl extraction, K^+^ competes with NH_4_^+^ for exchangeable sites on the biochar surface; therefore, the low NH_4_^+^ release indicates that only a small fraction of nitrogen was retained by ion exchange. A low desorption of NH_4_^+^ may also occur when adsorption takes place through oxygen-containing functional groups on the biochar surface rather than through ion exchange or physisorption.^15^ The partial solubilization observed in water suggests that a fraction of these precipitates—particularly surface-associated or poorly crystallized forms—may dissolve under low ionic strength conditions.^43^ This result indicates a gradual nitrogen supply, consistent with the concept of a slow-release fertilizer. Furthermore, the low desorption observed in KCl reinforces the potential of biochar to reduce nutrient losses through leaching.

## 4 Conclusions

The production of biochar from swine manure proved to be a promising strategy for nutrient recovery and environmental impact mitigation. Among the evaluated conditions, biochar activated with MgCl_2_ at a 3:1 ratio and pyrolyzed at 800 °C exhibited the best performance. Adsorption kinetics indicated that phosphate uptake was best described by the Elovich model (R^2^ = 0.99), while ammonium followed pseudo-second-order kinetics (R^2^ = 0.90), suggesting distinct but predominantly chemisorption-controlled mechanisms. Isotherm analyses revealed a high maximum adsorption capacity for phosphate (q_m_ = 67.56 mg g^–1^; R^2^ = 0.97, Langmuir model) and a lower capacity for ammonium (q_m_ = 17.48 mg g^–1^), better described by the Freundlich model (R^2^ = 0.95). The underlying mechanisms involve chemical precipitation (formation of magnesium phosphates) and electrostatic interactions for phosphorus and ion exchange for ammonium, potentially involving struvite formation. Finally, desorption assays validated the material’s slow-release potential, showing negligible phosphorus release in deionized water and a gradual release of nitrogen. Therefore, optimizing activation and carbonization with MgCl_2_ at 800 °C represents a viable route for producing multifunctional biochars capable of integrating swine waste valorization with sustainable nutrient recovery in agricultural systems.

## Supplementary Material



## Data Availability

The data supporting the findings of this study are available within the article and its Supporting Information.

## Notes

Phosphorus release test results (Table S1); proximate analysis of raw swine manure (Table S2); elemental and mineral composition of raw swine manure determined by EDS (Table S3) (PDF)
