# Supplementary material for: Biochar from Swine Manure: An Alternative for Nutrient Recovery and Slow-Release Fertilization
Source: ACS Omega. 2026 Feb 25;11(9):15201–12. doi: 10.1021/acsomega.5c12363 (PMC12980259; doi:10.1021/acsomega.5c12363)
Supplement: Supplementary file 1 [file ao5c12363_si_001.pdf]

Supporting Information

BIOCHAR FROM SWINE MANURE: AN ALTERNATIVE FOR NUTRIENT RECOVERY AND SLOW-RELEASE FERTILIZATION

Authors: Larissa Almeida Nascimento[a]; André Pereira Rosa[a*]; Augusto Vilela França[a]; Rita de Cássia Superbi de Sousa[b]; Renata Pereira Lopes Moreira[b];

[a]Department of Agricultural Engineering, Federal University of Viçosa, Viçosa, MG 36570-900, Brazil

[b]Department of Chemical Engineering, Federal University of Viçosa, Viçosa, MG 36570-900, Brazil

*Corresponding author.
E-mail address: andrerosa@ufv.br

- Number of pages: 1

- Number of tables: 1

RESULTS AND DISCUSSION

Table S1. Phosphorus Release Test

Treatmeant	P-PO_4^{3-} [mg L^{-1}]
BC 800	3.67 (± 0.22)
BC 400	8.26 (±0.06)
BC 800-$MgCl_2$ (3:1)	-0.059 (< LQ)

The water extraction test revealed distinct differences in the stability of phosphorus among the evaluated biochars. The unmodified materials released measurable amounts of P into solution, with BC400 showing the highest concentration (8.26 ± 0.06 mg L^{-1}) and BC800 releasing significantly less (3.67 ± 0.22 mg L^{-1}). This decrease with increasing pyrolysis temperature is consistent with the greater mineral stability and reduced solubility of P-bearing phases formed at higher carbonization temperatures [1].

REFERENCES

[1] R. Li *et al.*, "Enhancing phosphate adsorption by Mg/Al layered double hydroxide functionalized biochar with different Mg/Al ratios", *Sci. Total Environ.*, vol. 559, p. 121–129, jul. 2016, doi: 10.1016/J.SCITOTENV.2016.03.151.
